# Supplementary material for: Lysophosphatidic Acid Accelerates Bovine In Vitro-Produced Blastocyst Formation through the Hippo/YAP Pathway
Source: Int J Mol Sci. 2021 May 31;22(11):5915. doi: 10.3390/ijms22115915 (PMC8198578; doi:10.3390/ijms22115915)
Supplement: Supplementary file 1 [file ijms-22-05915-s001.zip › ijms-1217136-supplementary.pdf]

Supplementary Table S1. List of primers used for quantitative RT-PCR. Sense and anti-sense sequences are indicated by 's' and 'as' respectively. Ta = annealing temperature.

Gene	NCBI	Sequence (5′-3′)	Sense	Ta (°C)
CDX2	NM_001206299.1	AACCTGTGCGAGTGGA	s	60
		GCGACTGTAGTGAAACTCC	as	
OCT4	NM_174580.3	TAGCCACATCGCCCAGCAGC	s	60
.		GAAAGGAGACCCAGCAGCCTCA	as	
RPL15	NM_001077866.1	CACAAGTTCCACCACACTATTGG	s	61
		TGGAGAGTATTGCGCCTTCTC	as	
SDHA	NM_174178.2	GCAGAACCTGATGCTTTGTG	s	64
		CGTAGGAGAGCGTGTGCTT	as	
SOX2	NM_001105463	CCATGCAGGTTGACATCGT	s	60
		ACACAACTACGGAAACTAAAAGTGG	as	
TAZ	NM_001193047.1	AGATGACCTTTACGGCCACTG	s	60
		TTCCTAGGGTCTTGCCATGTG	as	
TEAD4	XM_010805630.3	GGCAAGATGTATGGTCGGAA	s	61
		TGCCTGATCCTTTAGCTTGG	as	
YAP	XM_024975708.1	TCCTTTGAGATCCCTGACGATG	s	53
		TGACGTTCATCTGGGAGAGC	as	
YWHAZ	XM_025001429.1	GCATCCCACAGACTATTTCC	s	56
		GCAAAGACAATGACAGACCA	as	
